# Observation of renal function after multiple DE-MRI examinations in atrial fibrillation ablation patients without kidney diseases

**DOI:** 10.1186/1532-429X-13-S1-P254

**Published:** 2011-02-02

**Authors:** Christian Mahnkopf, Manfred Duecker, Stefan Holzmann, Guido Ritscher, Oliver Turschner, Helge Simon, Johannes Brachmann, Anil M Sinha

**Affiliations:** 1 Klinikum Coburg, Coburg, Germany

## Background

Gadolinium-based MRI examinations have become essential for the treatment of patients with atrial fibrillation (AF). Gadolinium-based contrast agents can trigger nephrogenic systemic fibrosis (NSF).

## Objective

We thought to analyze the renal function in patients with normal and mildly reduced renal function after performing multiple MRI examinations prior to and following an AF ablation procedure.

## Methods

MRI was performed in 40 patients (27 males, age 62±9 years) who were scheduled for pulmonary vein isolation (PVI) and had a glomerularfiltration rate (GFR) of >30ml/min. A total of three MRIs were acquired prior to, directly after and 24 hours after PVI, using weight-dependent dosages of gadolinium pententat (Gd-DPTA). 46,8±14,9 ml Gd-DPTA was given within a 3±1 day period. Renal function [defined as serum creatinine, blood urea nitrogen (BUN) and GFR] was measured before, acutely after and 12±2 weeks after the procedure. During follow-up, patients were examined for symptoms of NSF.

## Results

No signs of significant reductions in renal function were observed. In addition, during a follow-up period of 15±3 weeks, no patient presented with early signs of NSF. Table 1.

**Table 1:** Course of renal function after multiple MRI

	Before PVI	Acute after PVI	Long Term after PVI	p-value
Creatine	1.1 ± 0.2	1.02 ± 0.2	1.0 ± 0.1	n.s.
BUN (mg/dl)	18.8 ± 6.6	15.2 ± 5.6	15.8 ± 4.4	n.s.
GFR	75.1 ± 19.2	80.7 ± 21.7	76.1 ± 10.2	n.s.

## Conclusion

For optimal ablation treatment of AF, multiple gadolinium-based MRI examinations appeared to be safe in regards to renal function; therefore, multiple MRIs could be utilized in patients with normal or mildly reduced renal function.

